# Supplementary material for: Nanodomain poling unlocking backward nonlinear light generation in thin film lithium niobate
Source: Nanophotonics. 2025 Dec 9;14(26):4729–37. doi: 10.1515/nanoph-2025-0429 (PMC12714043; doi:10.1515/nanoph-2025-0429)
Supplement: Supplementary file 1 — Supplementary Material Details [file j_nanoph-2025-0429_suppl_001.zip › logos/dg-degruyter.pdf]

DE GRUYTER
