# Supplementary figures and images for: Nanodomain poling unlocking backward nonlinear light generation in thin film lithium niobate
Source: Nanophotonics. 2025 Dec 9;14(26):4729–37. doi: 10.1515/nanoph-2025-0429 (PMC12714043; doi:10.1515/nanoph-2025-0429)

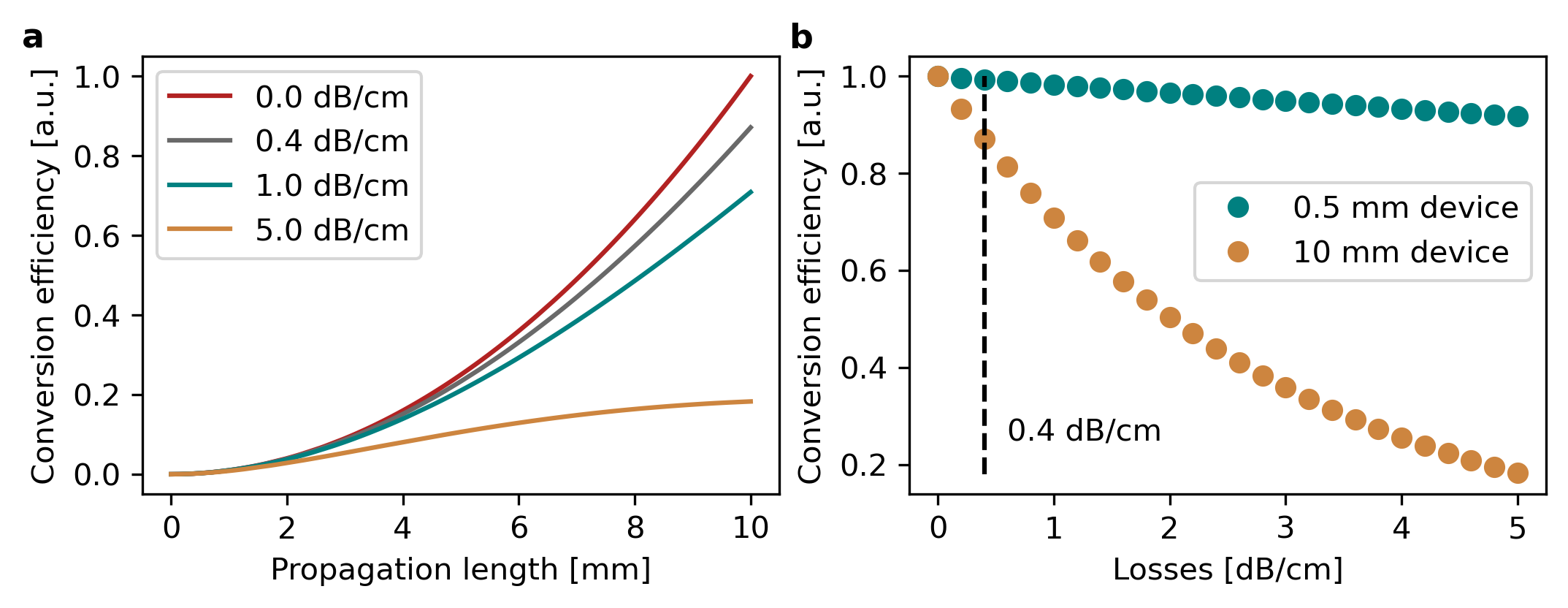

Supplement: Supplementary file 1 — Supplementary Material Details [file j_nanoph-2025-0429_suppl_001.zip › Figures_S/Losses_influence.png]

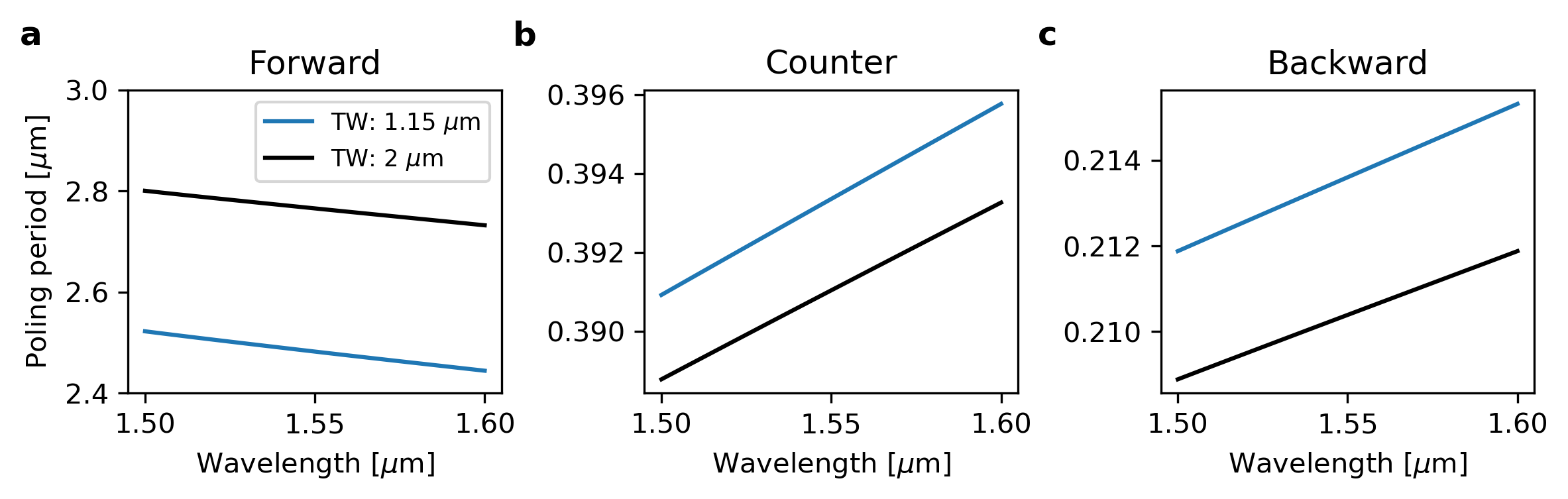

Supplement: Supplementary file 1 — Supplementary Material Details [file j_nanoph-2025-0429_suppl_001.zip › Figures_S/Periods.png]

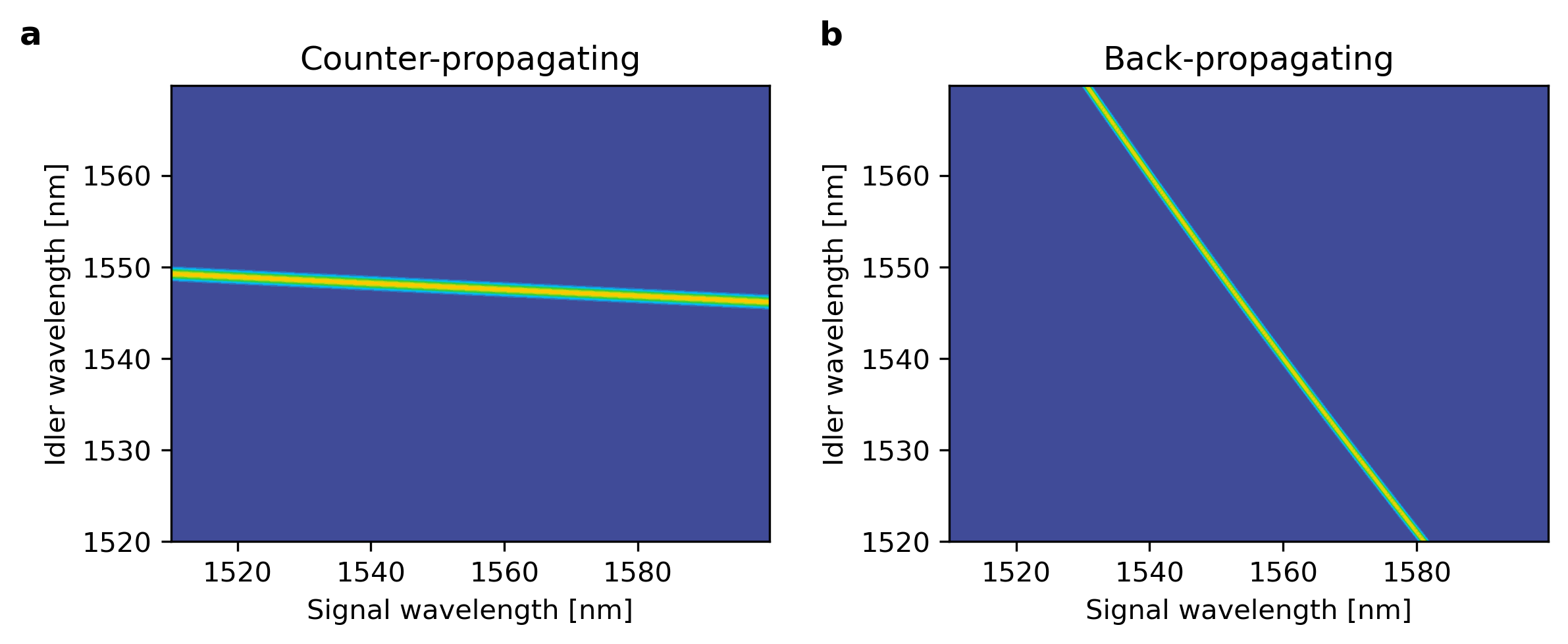

Supplement: Supplementary file 1 — Supplementary Material Details [file j_nanoph-2025-0429_suppl_001.zip › Figures_S/SFG_suppl.png]

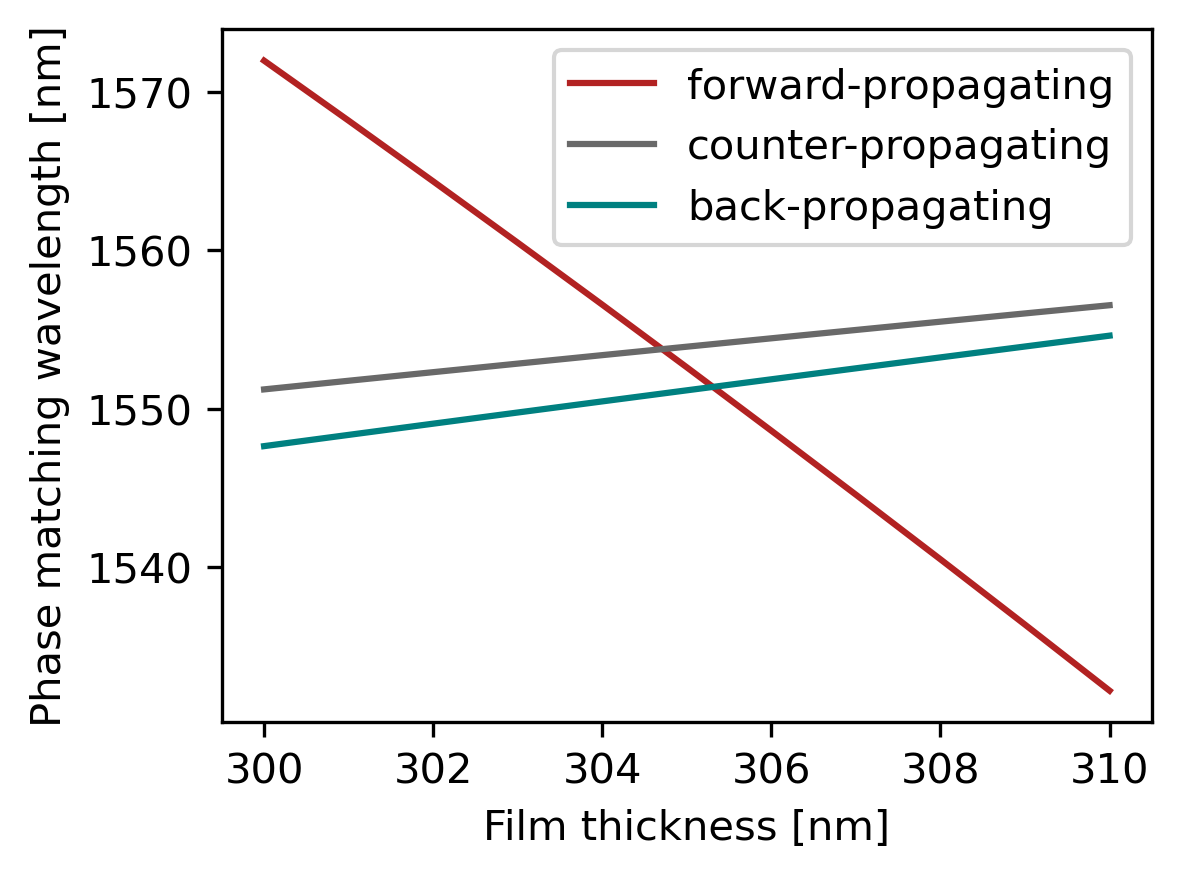

Supplement: Supplementary file 1 — Supplementary Material Details [file j_nanoph-2025-0429_suppl_001.zip › Figures_S/Phase_matching_thickness.png]

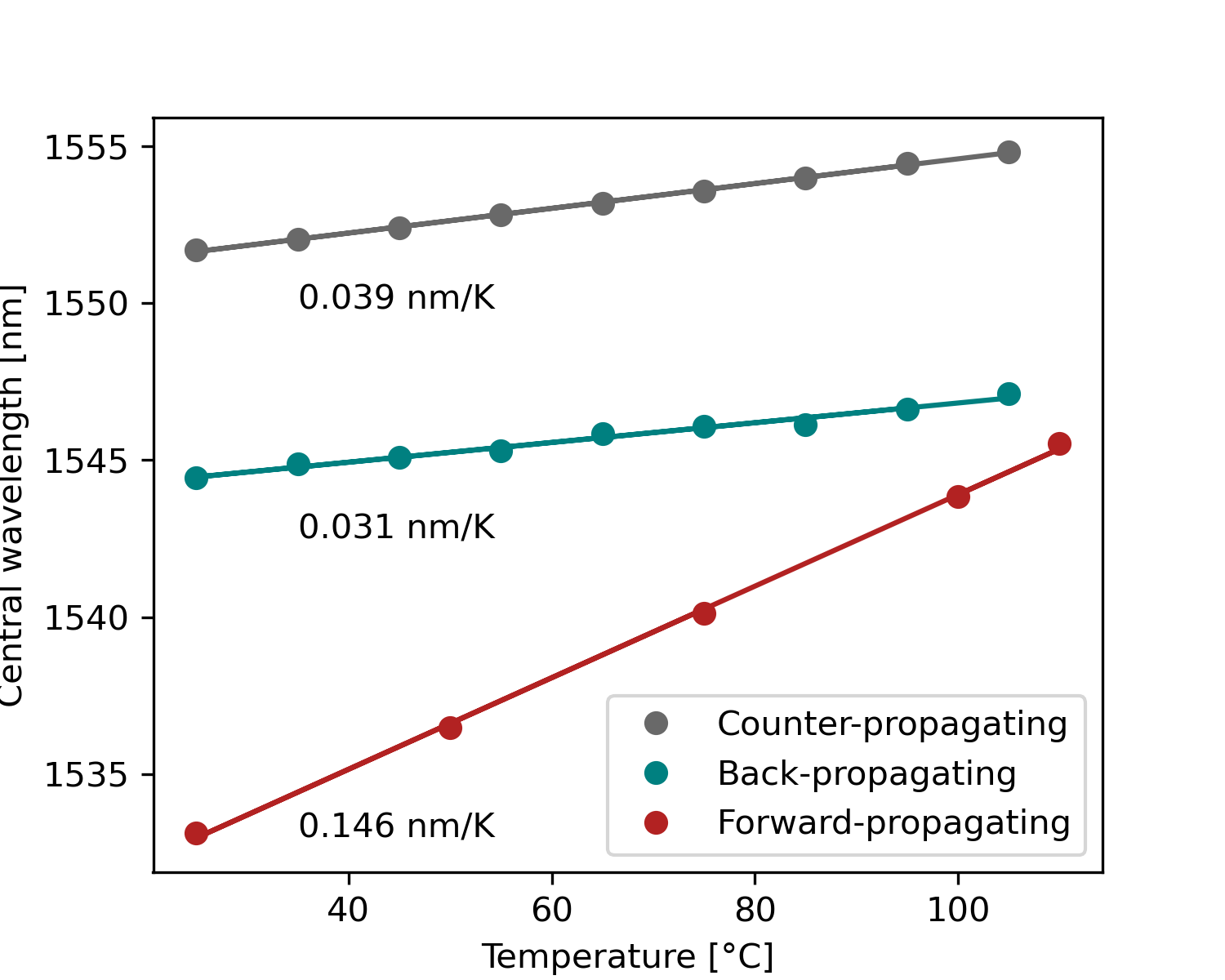

Supplement: Supplementary file 1 — Supplementary Material Details [file j_nanoph-2025-0429_suppl_001.zip › Figures_S/Temperature_tuning.png]

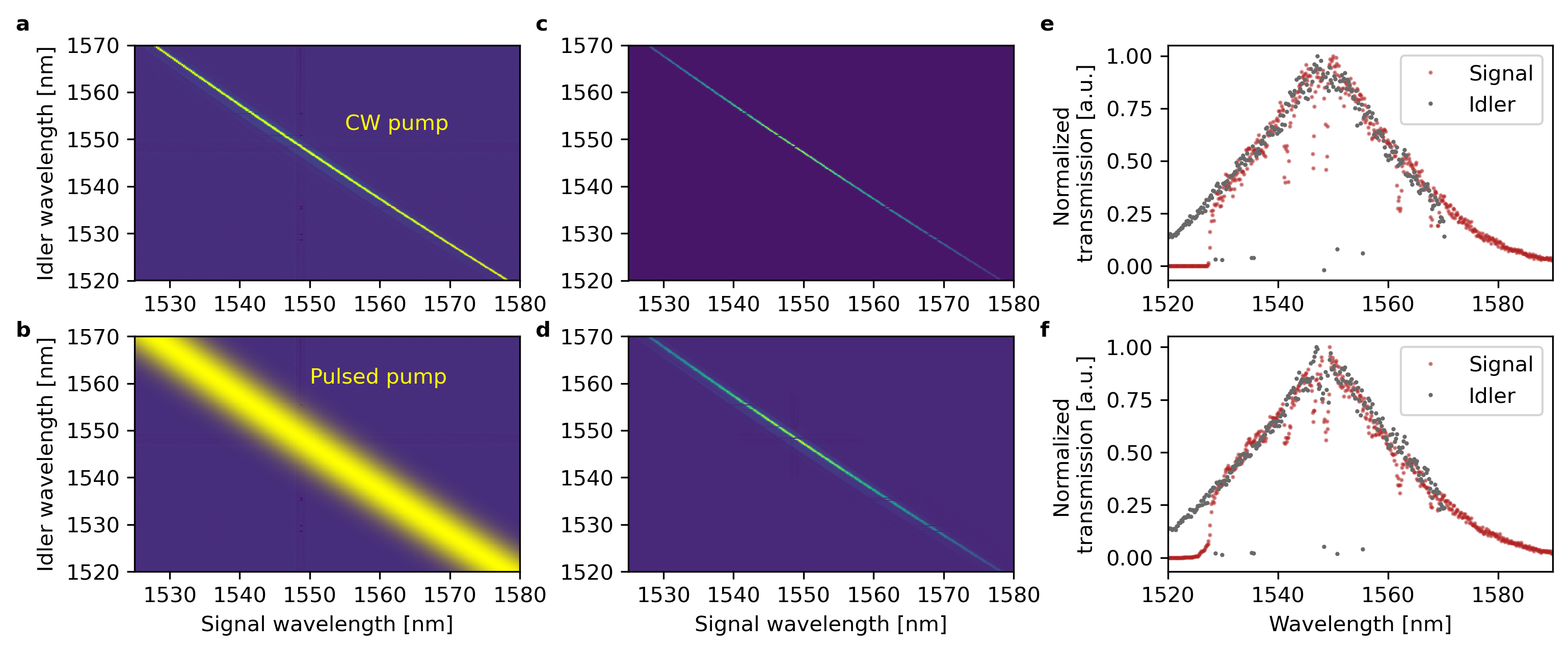

Supplement: Supplementary file 1 — Supplementary Material Details [file j_nanoph-2025-0429_suppl_001.zip › Figures_S/BP_bandwidth.png]

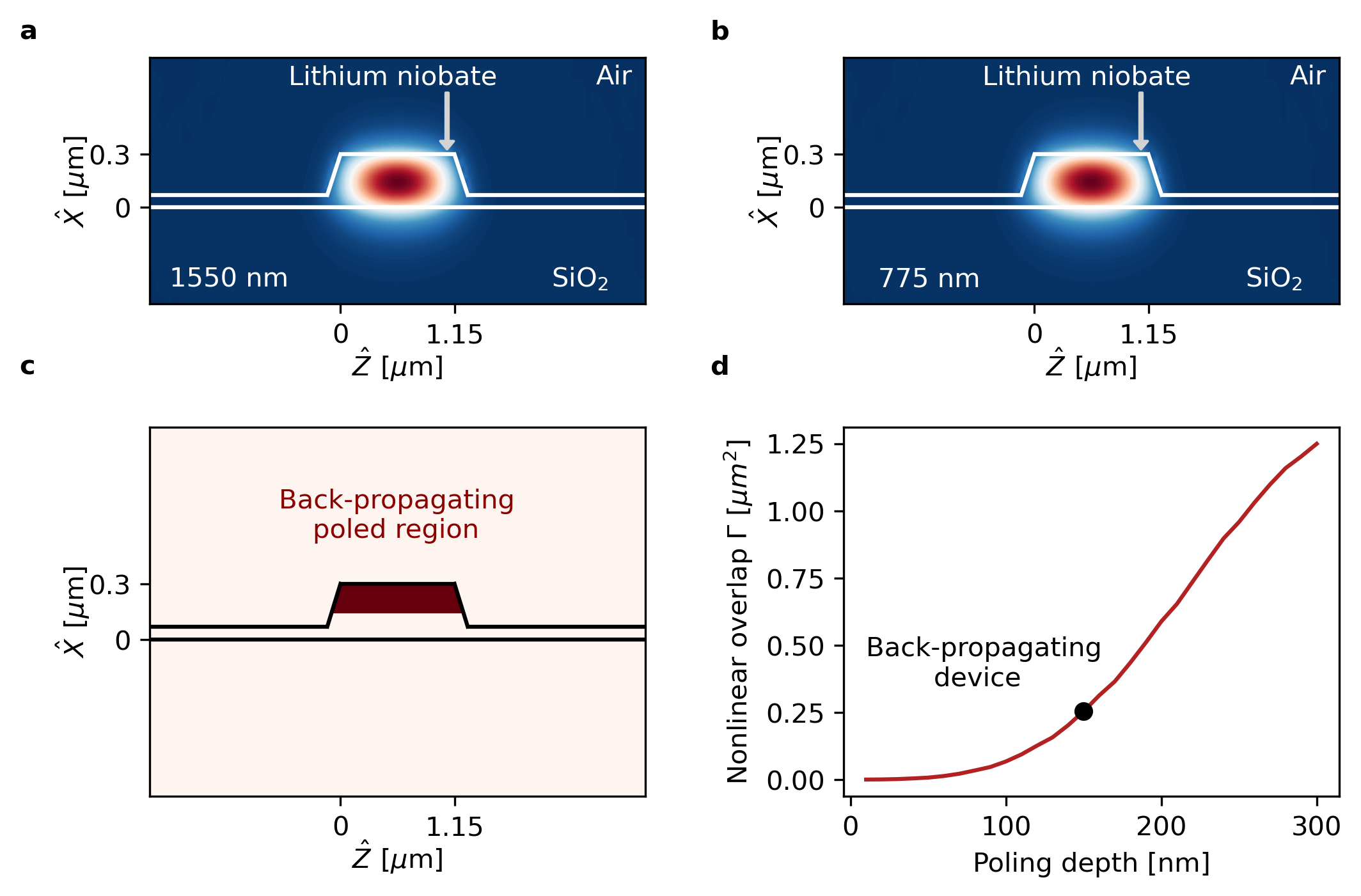

Supplement: Supplementary file 1 — Supplementary Material Details [file j_nanoph-2025-0429_suppl_001.zip › Figures_S/Modes_1.15um_overlap.png]

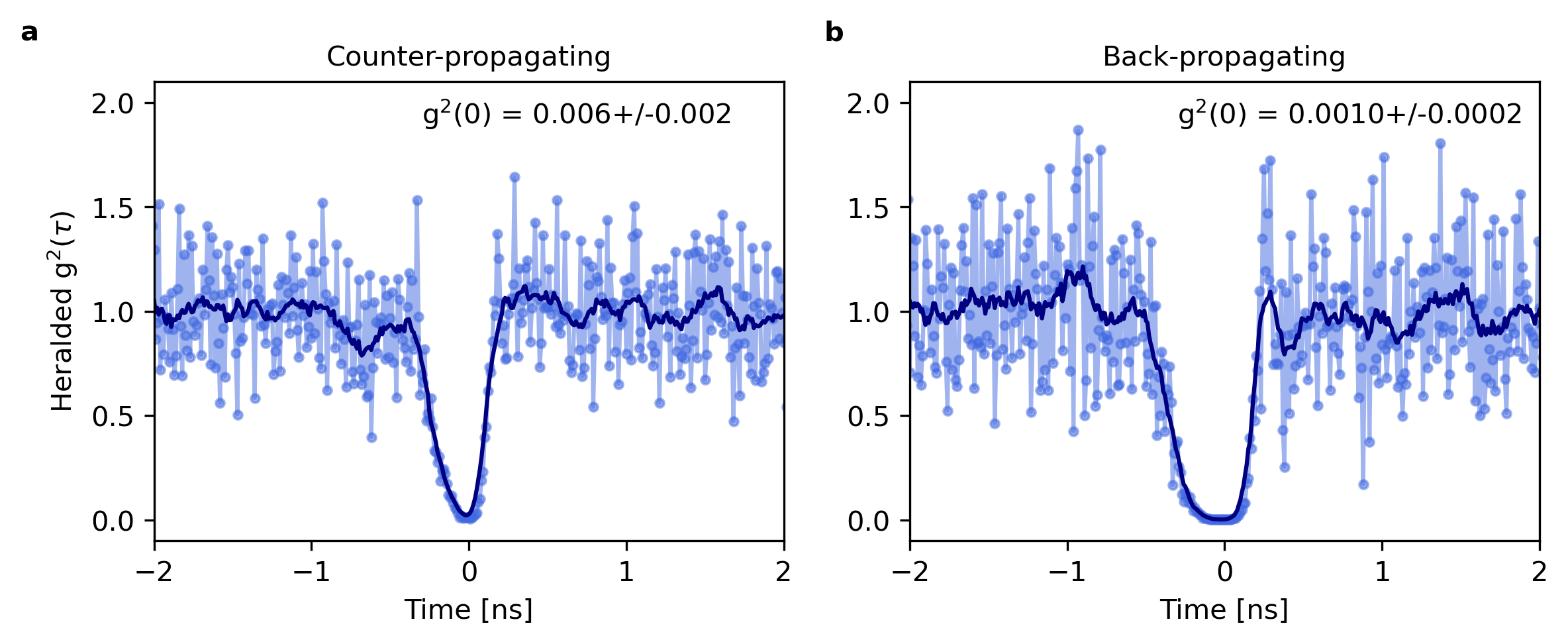

Supplement: Supplementary file 1 — Supplementary Material Details [file j_nanoph-2025-0429_suppl_001.zip › Figures_S/g2.png]

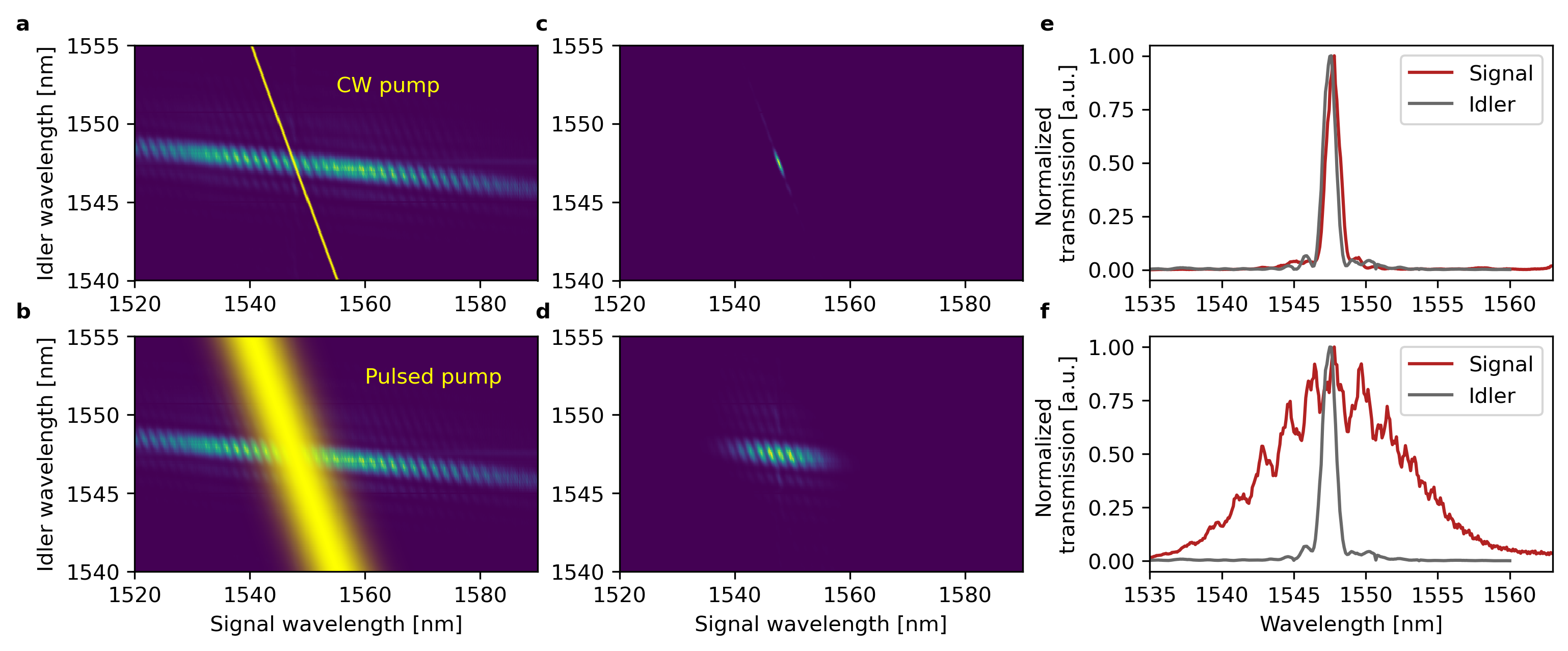

Supplement: Supplementary file 1 — Supplementary Material Details [file j_nanoph-2025-0429_suppl_001.zip › Figures_S/CP_bandwidth.png]

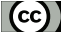

BY

Supplement: Supplementary file 1 — Supplementary Material Details [file j_nanoph-2025-0429_suppl_001.zip › logos/by.pdf]

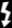

Supplement: Supplementary file 1 — Supplementary Material Details [file j_nanoph-2025-0429_suppl_001.zip › logos/DG_attention.pdf]

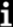

Supplement: Supplementary file 1 — Supplementary Material Details [file j_nanoph-2025-0429_suppl_001.zip › logos/DG_information.pdf]

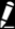

Supplement: Supplementary file 1 — Supplementary Material Details [file j_nanoph-2025-0429_suppl_001.zip › logos/DG_exercise.pdf]

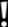

Supplement: Supplementary file 1 — Supplementary Material Details [file j_nanoph-2025-0429_suppl_001.zip › logos/DG_notice.pdf]

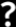

Supplement: Supplementary file 1 — Supplementary Material Details [file j_nanoph-2025-0429_suppl_001.zip › logos/DG_question.pdf]

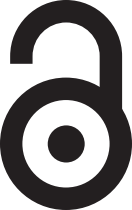

Supplement: Supplementary file 1 — Supplementary Material Details [file j_nanoph-2025-0429_suppl_001.zip › logos/Open_Access.pdf]
