# Supplementary material for: Nanodomain poling unlocking backward nonlinear light generation in thin film lithium niobate
Source: Nanophotonics. 2025 Dec 9;14(26):4729–37. doi: 10.1515/nanoph-2025-0429 (PMC12714043; doi:10.1515/nanoph-2025-0429)
Supplement: Supplementary file 2 — Supplementary Material Details [file j_nanoph-2025-0429_suppl_002.pdf]

Alessandra Sabatti*, Jost Kellner, Robert J. Chapman, and Rachel Grange

Supplementary material: Nanodomain poling unlocking backward nonlinear light generation in thin film lithium niobate

1 Optical modes and effect of partial poling depth

Waveguide cross sections with optical mode profiles for the C-band and visible wavelengths are reported in Fig.1 a-b. The waveguide represented has a top-width of 1.15 µm and is used for periodic poling with 215 nm period, corresponding to back-propagating phase matching. Unlike for the counter-propagating case, for which the used waveguide has a top width of 2 µm, for the back-propagating case the width was reduced to avoid excessive lateral growth of the domains.

We considered the nonlinear overlap factor Γ between the fundamental and second harmonic modes. We studied the effect of partial poling in the waveguide, to estimate its contribution to efficiency reduction for the case of back-propagating phase matching reported in the manuscript. The nonlinear overlap factor is expressed as follows [1]:

$$\Gamma = \frac{\left| \int_{\mathrm{LN}} p(x,z) \cdot (E^*_{z,\mathrm{FH}})^2 E_{z,\mathrm{SH}} \, dx \, dz \right|^2}{\left(\int_{\mathrm{all}} |E_{\mathrm{FH}}|^2 \, dx \, dz \right) \left(\int_{\mathrm{all}} |E_{\mathrm{SH}}|^2 \, dx \, dz \right)} \tag{1}$$

where p(x,z) represents the extension of the domains in the waveguide cross section. In Fig. 1 c it is represented the region of the waveguide that contributes to phase matching for the back-propagating device, with an estimated poling depth of 150 nm. In Fig. 1d it is shown how Γ increases with the increasing poling depth. For a device poled up until half of the film depth, 150 nm out of a total of 300 nm, like in the reported case of back-propagating phase matching, the overlap area is reduced to 20 % of the ideal value. The conversion efficiency depends linearly on the nonlinear overlap factor, and it is therefore reduced to the same percentage. Therefore a large part of the efficiency reduction for the back-propagating device can be attributed to the partial poling depth.

***Corresponding author: Alessandra Sabatti,** ETH Zurich, Department of Physics, Institute for Quantum Electronics, Optical Nanomaterial Group, CH-8093 Zurich, Switzerland, asabatti@ethz.ch; https://orcid.org/0009-0003-3233-5147. These authors contributed equally to this work.

Jost Kellner, ETH Zurich, Department of Physics, Institute for Quantum Electronics, Optical Nanomaterial Group, CH-8093 Zurich, Switzerland, kellnerj@ethz.ch; https://orcid.org/0000-0003-4037-7489. These authors contributed equally to this work.

Robert J. Chapman, ETH Zurich, Department of Physics, Institute for Quantum Electronics, Optical Nanomaterial Group, CH-8093 Zurich, Switzerland, rchapman@ethz.ch; https://orcid.org/0000-0002-0368-8483

Rachel Grange, ETH Zurich, Department of Physics, Institute for Quantum Electronics, Optical Nanomaterial Group, CH-8093 Zurich, Switzerland, grangera@ethz.ch; https://orcid.org/0000-0001-7469-9756

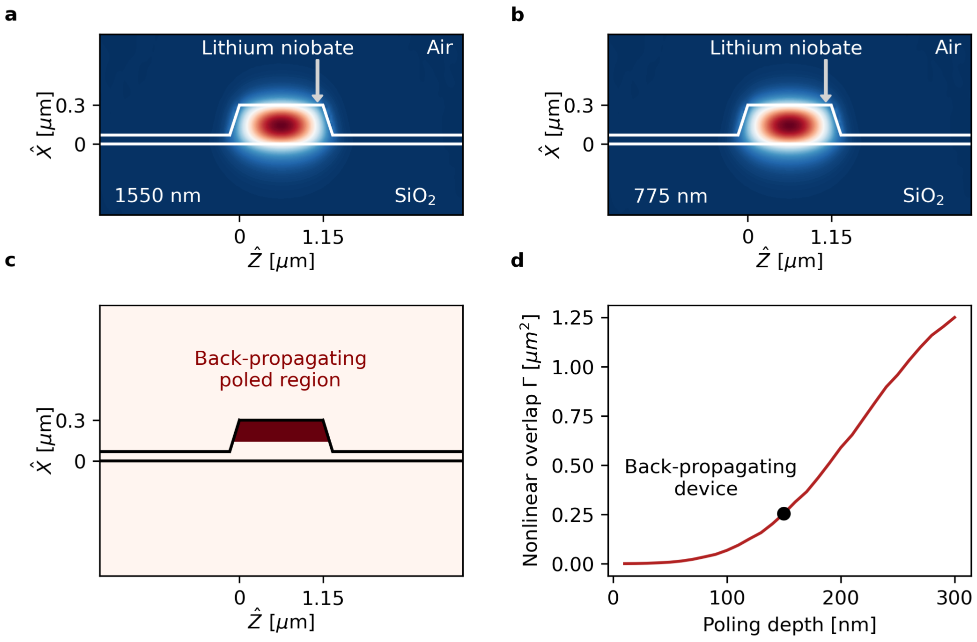

Fig. 1: Optical modes profiles and nonlinear overlap factor for back-propagating device. a-b Cross section and optical mode profiles for waveguide with $1.15\,\mu\mathrm{m}$ wide top width for **a** $1550\,\mathrm{nm}$ wavelength and **b** $775\,\mathrm{nm}$ wavelength. **c** Cross section and indication of poling depth for the back-propagating device. **d** Nonlinear overlap factor as a function of poling depth. With a poling depth such as in the back-propagating device the overlap factor is reduced to $20\,\%$ of the maximum value.

2 Periodic poling period

The periodic poling period for forward, counter- and backward-propagating phase matching is represented in Fig. 2. It is reported as a function of signal and idler wavelength, provided that they are degenerate. The blue and black curves indicate the different top widths used for periodic poling. Note that this data is obtained considering a thin film thickness of $300\,\mathrm{nm}$, and the periods used in the experiment were adapted to the real local film thickness to obtain phase matching close to $1550\,\mathrm{nm}$.

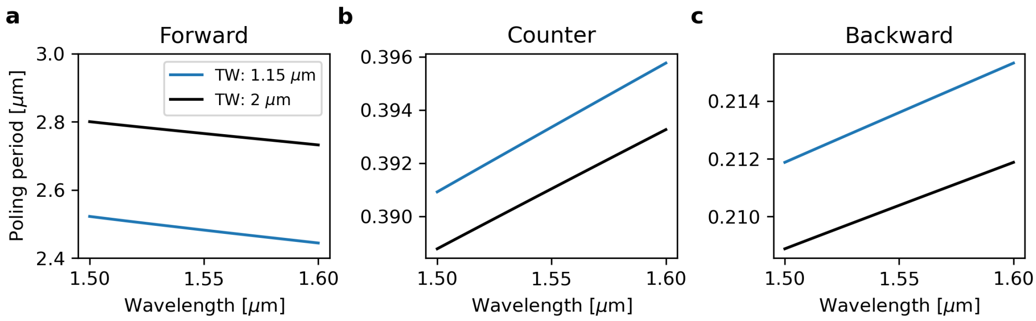

Fig. 2: Periodic poling periods for two different waveguide top widths for **a** forward-, **b** counter- and **c** backward-propagating phase matching. The blue and black curves represent the period for top width of $1.15\,\mu\mathrm{m}$ and $2\,\mu\mathrm{m}$ respectively. Abbreviations: TW: top width

3 Film thickness variation influence on phase matching

The simulation in Figure 3 illustrates how the phase-matching wavelength varies with changes in film thickness over a 10 nm range. The counter-propagating and back-propagating configurations exhibit significantly lower sensitivity to thickness variations compared to the forward-propagating case. The variation rate for the phase matching is -3.98 for the forward-, 0.53 for the counter- and 0.70 for the back-propagating. This reduced sensitivity is advantageous when integrating multiple sources on a single chip, as it simplifies aligning their operating wavelengths.

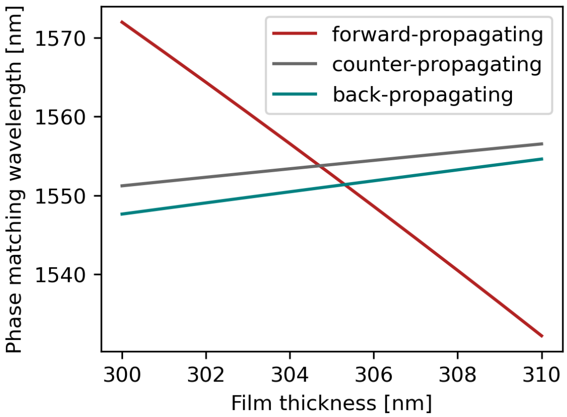

Fig. 3: Phase matching shift as a function of film thickness variation for the three phase matching configurations. The counter- and back-propagating devices exhibit a lower film thickness dependence than the forward-propagating.

4 Temperature tuning

The thermal tuning of both sources was characterized by measuring second harmonic generation with temperature ranging from 25 °C to 105 °C. The central phase matching wavelength as a function of temperature is reported in Fig. 4, for the counter-propagating and back-propagating device. The rates resulting from linear fits are 0.039 nm/K and 0.031 nm/K, indicating a similar moderate tuning rate for both sources. For comparison, we report the data from the second harmonic signal produced by a forward propagating device, characterised by a rate of 0.146 nm/K, which is significantly larger than the other two. This behavior is expected if we consider that there is a significant difference in the thermal coefficients of lithium niobate for light in the visible and in the near infrared, as reported by Moretti et al [2]. The phase mismatch for a forward-, counter- and backward-propagating device are respectively $\Delta k_f = 2\pi[n_P - n_S]/\lambda_P$, $\Delta k_c = 2\pi n_P/\lambda_P$ and $\Delta k_b = 2\pi[n_P + n_S]/\lambda_P$. Considering that the forward phase-matching has a term proportional to the refractive index difference, it is evident that tit will experience a larger tuning rate than the other two phase matching schemes.

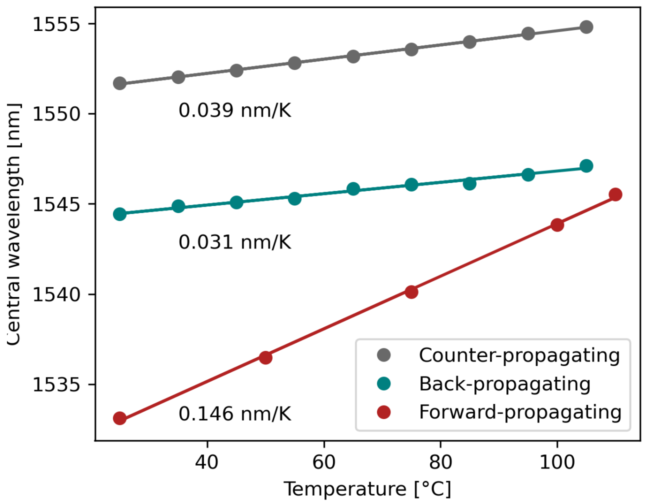

Fig. 4: Thermal tuning of quasi-phase matching. Second harmonic generation central wavelength as a function of sample temperature for a counter-propagating and a back-propagating source in blue and orange respectively. The thermal tuning of the second harmonic signal of a forward propagating device (green) is reported as a comparison.

5 Propagation losses effect on efficiency

We estimated the effect of propagation losses on the conversion efficiency in the process of second harmonic generation for both the fundamental and second harmonic light. Nonlinear regions of different lengths and different loss values were investigated. Fig. 5 a shows the conversion efficiency as a function of length for different loss values. Note that for simplicity losses are assumed to be the same for the fundamental and second harmonic. Fig. 5 shows a comparison of the conversion efficiency as a function of propagation loss for the device length we studied in the main manuscript, and for a 10 mm long device. While the short device efficiency is barely affected even by large losses, the long device shows a significant efficiency decay with losses. This has to be taken into consideration in the perspective of realizing a mirrorless optical parametric oscillator on chip. We therefore checked the conversion efficiency with an estimated value for the propagation losses in our devices of 0.4 dB/cm and we obtained a reduction in conversion efficiency of only 13 % with respect to the ideal case.

6 Simulation of sum frequency generation

We simulate the process of sum frequency generation for counter-and back-propagating phase matching to compare it with the measurement reported in Fig.3e-f of the main manuscript. The phase matching functions are obtained by calculating, at each signal and idler wavelength, the phase mismatch Δk between signal, idler and the sum photon that satisfies energy conservation. The conversion efficiency is then calculated at each point and plotted in a 2-d map. In Fig.6-a the phase matching function presents a moderate negative slope, while the function in 6-b has a slope with value -1. These results are in excellent agreement with the measured data, both considering the width and the slope of the line. The larger intensity in the central region of the spectrum in the experimental data can be explained by the transmission function of the grating couplers for both near infrared and visible light, which present a maximum in the transmission at 1550 nm and 775 nm. Note that the back-propagating simulation, unlike the measured data, does not include the process of second harmonic, from the individual light sources, therefore the horizontal and vertical lines are not visible in this plot.

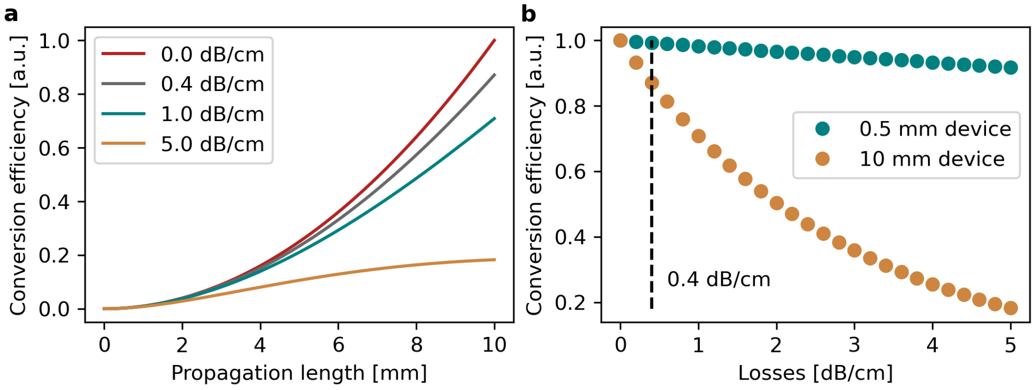

Fig. 5: Influence of propagation losses on conversion efficiency. a Conversion efficiency as a function of propagation length in a PPLN device for increasing loss values. Note that the loss value is assumed to be the same for the fundamental and second harmonic. **b** Conversion efficiency as a function of propagation loss for a $0.5\,\mathrm{mm}$ and a $10\,\mathrm{mm}$ device.

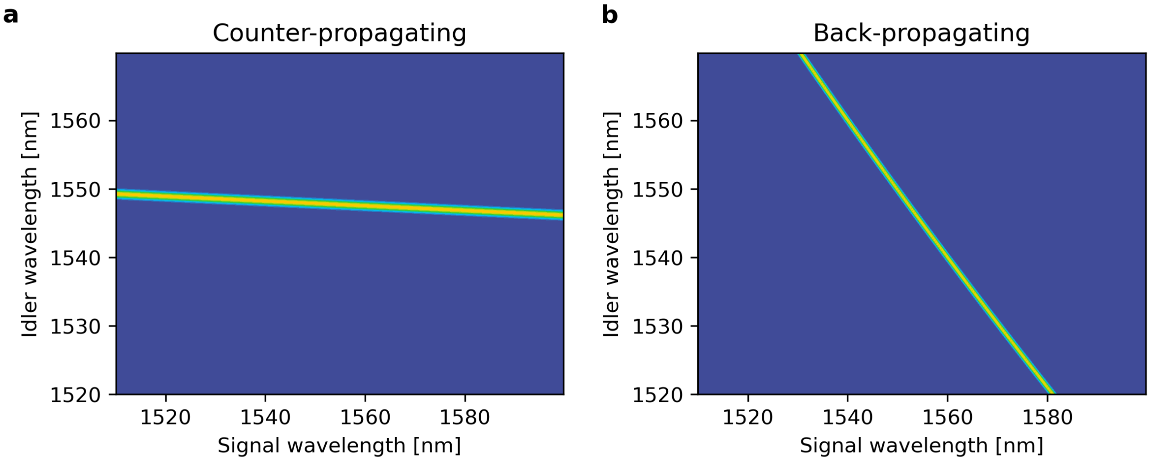

Fig. 6: Phase matching function obtained from the sum frequency of two light sources in the C-band for **a** counter- and **b** back-propagating signal and idler.

7 Estimate of photon bandwidth

The spectra of signal and idler generated by the spontaneous parametric down conversion process is estimated based on the sum frequency generation measurement, both for a continuous wave pump and for a pulsed pump, that have different bandwidths. The two considered pump profiles are overlapped with the sum frequency generation data, that correspond to the phase matching efficiency of the source as a function of signal and idler wavelength in Fig 7 a-b and 8 a-b. Fig 7 c-d and 8 c-d report the overlapped spectrum, that corresponds to the joint spectral intensity of the photon pairs produced by spontaneous parametric down-conversion. Lastly, the inferred spectrum of signal and idler photons is reported in panels e and f for both figures.

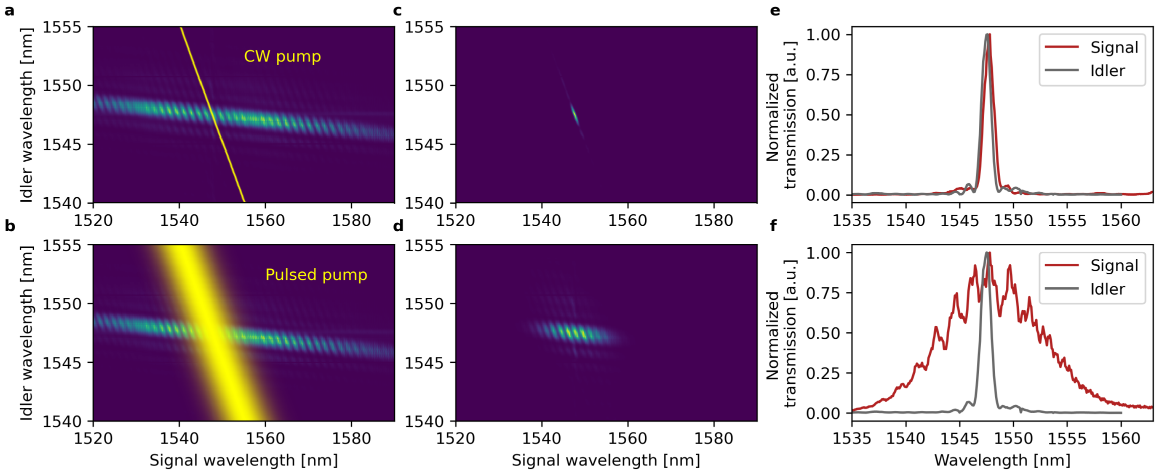

Fig. 7: Photon bandwidth estimate for counter-propagating SPDC. **a,b** Experimental phase matching function overlapped with the spectral shape of a **a** CW pump and **b** a pulsed pump. **c-d** Inferred joint spectral intensity. **e-f** Signal and idler spectra for **e** CW and **f** pulsed pump.

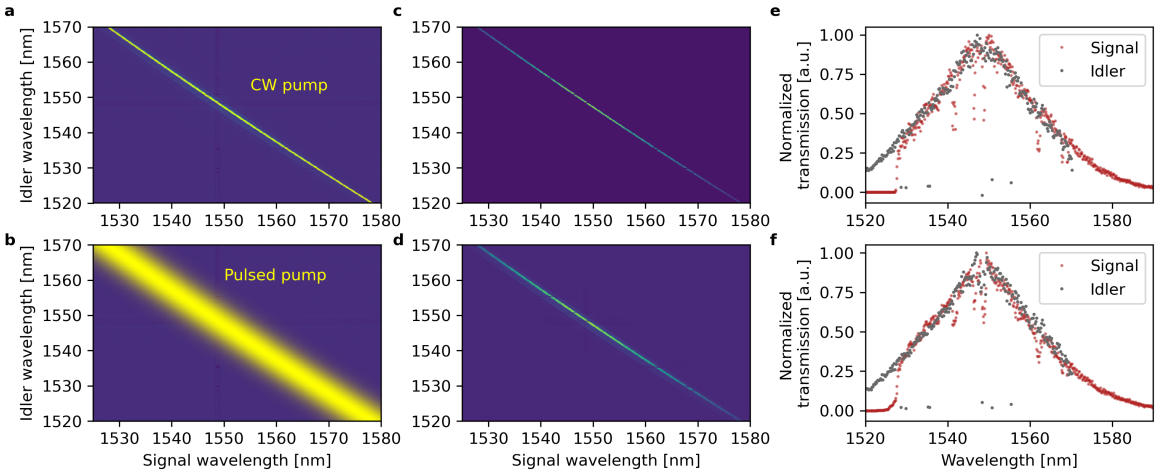

Fig. 8: Photon bandwidth estimate for back-propagating SPDC. **a,b** Experimental phase matching function overlapped with the spectral shape of a **a** CW pump and **b** a pulsed pump. **c-d** Inferred joint spectral intensity. **e-f** Signal and idler spectra for **e** CW and **f** pulsed pump.

For the counter-propagating case the bandwidth of the pump affects the shape of the joint spectral amplitude and also the bandwidth of the generated photons. For a CW pump, signal and idler photons are expected to both have a 1 nm bandwidth, whereas in the pulsed pump case, the idler remains almost identical, while the signal experiences a larger bandwidth, that has a strong dependence on the pump bandwidth. For the back-propagating case, the signal and idler photons are expected to be identical among each other, and not to be influenced by the pump bandwidth, as it perfectly overlap with the phase matching functions. We therefore expect very broad photons, as reported in Fig. 8 e-f. Note that the displayed bandwidth corresponds to the grating coupler transmission and the real photon bandwidth would be larger.

8 Heralded second order correlation function

To assess the suitability of the two SPDC sources as heralded single-photon sources, we measure the heralded second-order correlation function $g^{(2)}$. For both sources, we follow the procedure described in Refs. [3, 4].

For the counter-propagating source, we split the signal photon between two detectors using a beamsplitter and use the idler photon as the herald. The measured $g^{(2)}$ trace is shown in Fig. 9 a, and we obtain a value of $g^{(2)}(0) = 0.006 \pm 0.002$.

Because the back-propagating source is a degenerate SPDC source, the signal and idler photons cannot be distinguished and must be probabilistically separated. To address this, we use a first beamsplitter to tap off one photon as the heralding photon, and a second beamsplitter to measure the correlations of the remaining photons. The resulting $g^{(2)}(0)$ value is 0.0010 ± 0.0002, and the full temporal dependence is shown in Fig. 9 b.

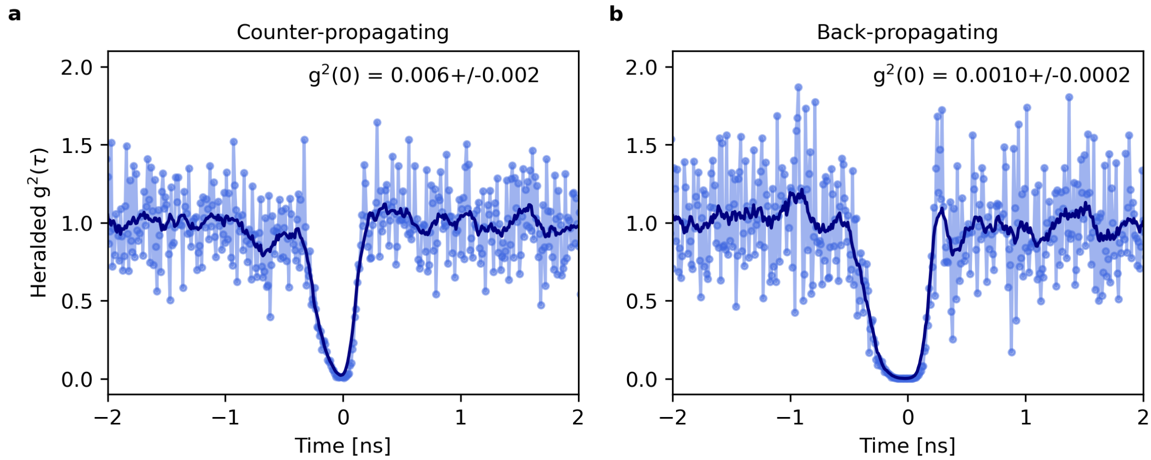

Fig. 9: Heralded second order correlation functions. $g^(2)(\tau)$ data and moving average for **a** counter-propagating and **b** back-propagating source.

References

[1] Xiaodong Shi, Sakthi Sanjeev Mohanraj, Veerendra Dhyani, Angela Anna Baiju, Sihao Wang, Jiapeng Sun, Lin Zhou, Anna Paterova, Victor Leong, and Di Zhu. Efficient photon-pair generation in layer-poled lithium niobate nanophotonic waveguides. *Light: Science & Applications*, 13(1):282, 2024.

[2] Luigi Moretti, Mario Iodice, Francesco G. Della Corte, and Ivo Rendina. Temperature dependence of the thermo-optic coefficient of lithium niobate, from 300 to 515 k in the visible and infrared regions. *Journal of Applied Physics*, 98(3):036101, 08 2005.

[3] Xiang Guo, Chang-ling Zou, Carsten Schuck, Hojoong Jung, Risheng Cheng, and Hong X Tang. Parametric down-conversion photon-pair source on a nanophotonic chip. *Light: Science &; Applications*, 6(5):e16249–e16249, November 2016.

[4] Stefano Bettelli. Comment on "coherence measures for heralded single-photon sources". *Physical Review A*, 81(3), March 2010.
